# Carboplatin and nab-paclitaxel chemotherapy with or without atezolizumab as front-line management for treatment-naïve metastatic nonsquamous non-small cell lung cancer with PD-L1 staining: a retrospective study

**DOI:** 10.1007/s00432-021-03873-3

**Published:** 2022-01-01

**Authors:** Bo Xu, Huihui Cheng, Kunhong Li, Yukai Lv, Xianshang Zeng, Tao Liu, Weiguang Yu, Wenbo Guo

**Affiliations:** 1 Department of Thoracic Surgery, The First Affiliated Hospital, Sun Yat-Sen University, No. 58, Zhongshan 2nd Road, Yuexiu District, Guangzhou, 510080 China; 2 Department of Anesthesiology, Renmin Hospital of Wuhan University, Wuhan, 430060 Hubei China; 3 Department of Anesthesiology, Wuhan Fourth Hospital, Puai Hospital, Tongji Medical College, Huazhong University of Science and Technology, No. 3, Gutian Road, Qiaokou District, Wuhan, 430000 China; 4 Department of Pediatrics, The First Affiliated Hospital, Sun Yat-Sen University, No. 58, Zhongshan 2nd Road, Yuexiu District, Guangzhou, 510080 China; 5 Department of Orthopaedics, The First Affiliated Hospital, Sun Yat-Sen University, No. 58, Zhongshan 2nd Road, Yuexiu District, Guangzhou, 510080 China; 6 Department of Intensive Care Unit, Wuhan Fourth Hospital, Puai Hospital, Tongji Medical College, Huazhong University of Science and Technology, No. 3, Gutian Road, Qiaokou District, Wuhan, 430000 China; 7 Department of Invasive Technology, The First Affiliated Hospital, Sun Yat-Sen University, No. 58, Zhongshan 2nd Road, Yuexiu District, Guangzhou, 510080 China; 8 Puai Hospital, Tongji Medical College, Huazhong University of Science and Technology, No. 3, Gutian Road, Qiaokou District, Wuhan, 430000 China

**Keywords:** Atezolizumab, Carboplatin, Non-small cell lung cancer, Chemotherapy, Programmed cell death-ligand 1

## Abstract

**Purpose:**

The aim of this retrospective review was to compare the efficacy and safety of the atezolizumab plus carboplatin and nab-paclitaxel regimen versus the carboplatin and nab-paclitaxel regimen as front-line management for treatment-naïve, metastatic nonsquamous programmed death-ligand 1 (PD-L1)-positive non-small cell lung cancer (NSCLC) in a selected population.

**Methods:**

Consecutive patients with untreated, metastatic nonsquamous PD-L1-positive NSCLC who initially received the atezolizumab plus carboplatin and nab-paclitaxel (ACN) regimen or carboplatin and nab-paclitaxel (CN) regimen were retrospectively identified in two medical institutions from 2017 to 2020. The co-primary end points were overall survival (OS) and progression-free survival (PFS); secondary end point was the rate of key adverse events (AEs).

**Results:**

In sum, 171 patients were retrospectively analysed, 47 of whom were excluded according to the criteria used in this study, leaving 124 patients (ACN: *n* = 60, median age 64 years [range 46–75]; CN: *n* = 64, 63 years [47–72]). The median duration of follow-up was 27 months [range 1–37]. At the final follow-up, the median OS was 19.9 months (95% confidence interval [CI], 16.3–22.5) in the ACN group vs. 14.8 months (95% CI 12.5–17.2) in the CN group (hazard ratio [HR] 0.51, 95% CI 0.33–0.77; *p* = 0.001). A marked distinction in the median PFS was seen (8.5 months [95% CI 6.7–9.4] in the ACN group vs. in the CN group [5.1 months [95% CI 3.6–6.8; HR 0.60; 95% CI 0.38–0.95; *p* = 0.005]). The rates of the key AEs (neutropenia and anaemia) were greater in the ACN group than in the CN group (all *p* < 0.05), but these AEs were manageable.

**Conclusion:**

Among selected populations of individuals with treatment-naïve, metastatic nonsquamous PD-L1-positive NSCLC, atezolizumab combined with carboplatin and nab-paclitaxel chemotherapy might have encouraging anticancer activity, with a tolerable safety profile.

## Introduction

Non-small cell lung cancer (NSCLC) is the most common type of lung cancer, accounting for approximately 90% of patients, and that proportion is expected to continue to climb (Gettinger et al. 2016; Horn et al. 2017). Some patients tend to have an increased risk of metastatic nonsquamous NSCLC (Gettinger et al. 2015; Ready et al. 2019). Regardless of aetiology, in patients with metastatic nonsquamous NSCLC for whom available treatment options are lacking (Antonia et al. 2019; Horn et al. 2017), current front-line therapies for metastatic nonsquamous NSCLC are restricted by previous FDA-approved guidelines, and prognosis remains poor, with a median overall survival (OS) of about 10 months (Herbreteau et al. 2020; Mansfield et al. 2020). However, the landscape for metastatic nonsquamous NSCLC has been improving because ground-breaking, evidence-based treatments are increasingly accessible (Hida et al. 2018; Rittmeyer et al. 2017). Despite improvements in management, the 5-year survival rate for metastatic nonsquamous NSCLC varies from 2 to 30% (West et al. 2019). Differentiated management modalities for these patients seem to have become a crucial aspect of improving survival, but updates to metastatic nonsquamous NSCLC management concepts initiated by cancer immunotherapy research are still pending(Reck et al. 2020; West et al. 2019). So-called extended criteria with numerous immune checkpoint inhibitors have exhibited an encouraging clinical benefit of more than 1-year survival in patients with programmed cell death 1 (PD-1)-positive tumours (Reck et al. 2020), although currently, complementary approaches to metastatic nonsquamous NSCLC treatment are still in development (West et al. 2019).

Atezolizumab, an anti-PD-L1, humanised, engineered immunoglobulin G1 monoclonal antibody designed to block the binding of tumour cell-expressed programmed death-ligand 1 (PD-L1) to PD-1 and CD80 (B7.1), activates the immune response directed by T cells during tumour cell recognition and reverses the PD-L1/PD-1-mediated inhibition of T cells to reconstruct anticancer immunity by improving vascular endothelial growth factor (VEGF)-mediated immunomodulatory effects and chemotherapy-induced tumour cell antigen exposure (Furuya et al. 2021; Reck et al. 2020). Furthermore, combining atezolizumab with chemotherapy tend to have a synergistic effect in patients with NSCLC (Horn et al. 2018; Peters et al. 2017; Reck et al. 2020). The POPLAR trial(Fehrenbacher et al. 2016) and OAK trial (Rittmeyer et al. 2017) have indicated promising anticancer activity in pretreated metastatic nonsquamous PD-1-positive NSCLC, with median OS times of 12.6 months (95% confidence interval [CI] 9.7–16.4) and 13.8 months (11.8–15.7), respectively. However, to date, whether combining atezolizumab with cytotoxic chemotherapy can yield improved survival in individuals diagnosed with metastatic nonsquamous PD-1-positive NSCLC has remained unclear. Of note, the National Comprehensive Cancer Network (NCCN) management guiding principles have recommended immunotherapy-based combination regimens as front-line management for individuals with advanced NSCLC without epidermal growth factor receptor (EGFR) or anaplastic lymphoma kinase (ALK) mutations (Ettinger et al. 2019). Unfortunately, published data on the effect of immunotherapy-based combination regimens in Chinese patients are lacking. Hence, we launched a multicentre retrospective study to compare the efficacy and safety of atezolizumab in combination with carboplatin and nab-paclitaxel regimen versus carboplatin and nab-paclitaxel regimen as front-line management for treatment-naïve stage IV nonsquamous PD-L1-positive NSCLC in selected populations of Chinese patients.

## Materials and methods

### Study design and patient eligibility

Between June 1, 2017, and June 30, 2020, consecutive individuals with treatment-naïve metastatic nonsquamous PD-L1-positive NSCLC who underwent front-line therapy with the atezolizumab plus carboplatin and nab-paclitaxel (ACN) regimen or carboplatin and nab-paclitaxel (CN) regimen in two medical centres (the First Affiliated Hospital, Sun Yat-sen University and the Tongji Medical College, Huazhong University of Science and Technology) and for whom patient characteristics were obtainable were retrospectively analysed. The following inclusion criteria were applied: clinically or histologically confirmed stage IV nonsquamous NSCLC; tumour cells expressing PD-L1 (tumour cells [TCs] or tumour-infiltrating immune cells [ICs] expressing PD-L1 ≥ 1%) (West et al. 2019); at least one cycle of front-line combined therapy; an Eastern Cooperative Oncology Group performance status score of 0 or 1; at least 1 calculable lesion per the modified response evaluation criteria in solid tumours (mRECIST); and adequate end-organ function (West et al. 2019). Major exclusion criteria included the following: a lack of patient characteristics; pretreated NSCLC (i.e. cytotoxic chemotherapy and/or radiotherapy, anti-PD-1 or anti-PD-L1 antibodies, or lung surgery); oncogenic driver mutations (i.e. EGFR or ALK mutations); discontinuity of front-line therapy, regardless of toxicity related to treatment; symptomatic central nervous system involvement; symptomatic deterioration attributed to disease progression per the mRECIST; contraindications listed in the drug leaflet; autoimmune-related diseases (i.e. systemic lupus erythaematosus, mandatory spondylitis, or autoimmune diabetes); bone marrow suppression; anaemia; and mental illness.

### Study design and management

A retrospective multicentre review was executed in which eligible individuals had experienced the ACN or CN regimen for metastatic nonsquamous PD-L1-positive NSCLC. The decision to manage the disease by adopting the ACN or CN regimen was rendered by the chief thoracic surgeon (BX). Atezolizumab (1200 mg, intravenously) and carboplatin (area under the curve, 6 mg/mL/min) were administered intravenously every 3 weeks (West et al. 2019). Nab-paclitaxel was administered intravenously at 100 mg/m^2^ every week (West et al. 2019). The same drug delivery schedule was used in each case. Three weeks were considered a treatment cycle. Drug-related therapy was administered until the occurrence of unbearable toxicity, loss of clinical benefit, progression as per mRECIST, or death. Continuation of drug delivery after progression was permitted during the follow-up period. The concomitant therapies used were similar in the two regimens.

### Outcomes and assessments

Tumour samples for PD-L1 testing were available from each patient. Immunohistochemistry analysis of PD-L1 expression on TCs and ICs was done with a PD-L1 IHC 28-8 pharmDx assay (Dako, Carpinteria, CA) as previously reported (Horn et al. 2017; Peters et al. 2017). TCs were categorised in accordance with pre-set values (1% ≤ TC1 < 5%, 5% ≤ TC1 < 50%, TC3 ≥ 50%) (West et al. 2019). PD-L1-positive NSCLC was defined as TC ≥ 1%. IC data were not required when recording baseline data. The co-primary end points were OS and progression-free survival (PFS). Tumour response was evaluated using contrast-enhanced CT unless contraindicated as per the mRECIST. OS and PFS were defined as the time from the beginning of front-line combined therapy to death from any cause and to progression or death from any cause, respectively. The secondary end point was the rate of major adverse events (AEs). AEs were graded as per National Cancer Institute Common Terminology Criteria for Adverse Events, version 5.0. Safety was evaluated as per major AEs reported during the entire treatment. Tumour response and progression were individually evaluated following the start of front-line combined therapy. Follow-up occurred every 3 weeks until insufferable toxicity or death, whichever occurred first.

### Statistical analysis

The OS and PFS after the beginning of front-line combined therapy were assessed between the two treatment groups with a log-rank test. The estimation of median survival was executed using the Kaplan–Meier approach. The follow-up time was estimated with the reverse Kaplan–Meier method. The hazard ratios (HRs) and 95% CIs for median survival were assessed using a Cox regression model with a Cox proportional hazards model, with age, sex, ECOG-PS, time since diagnosis, PD-L1 tumour expression, tobacco use history, brain metastasis, number of metastatic sites utilised as covariates, and intervention was provided as the time-dependent variable. A two-tailed *p* value < 0.05 was regarded as significant. We analysed data using SPSS 26.0 software (IBM, Inc., New York).

## Results

### Demographic characteristics

**Fig. 1:**
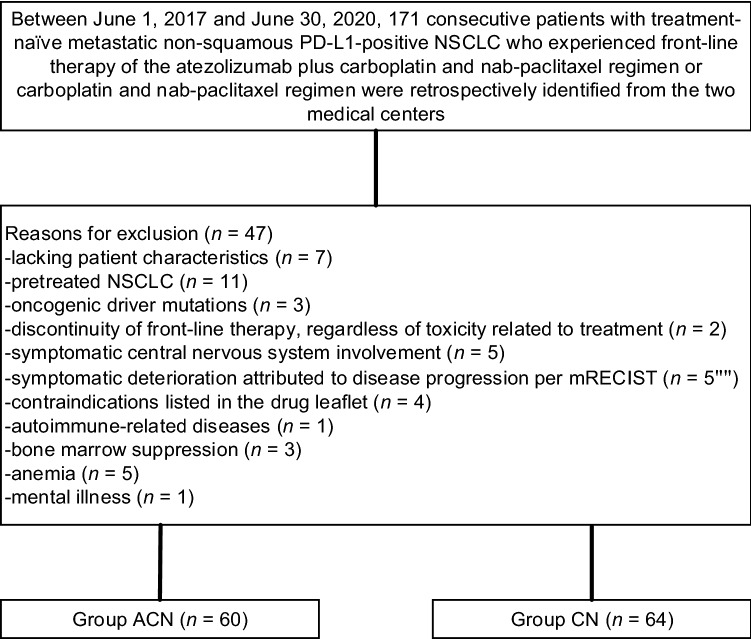
Flow diagram displaying the methods adopted to identify patients to assess the efficacy and safety of the ACN regimen versus CN regimen as front-line management for metastatic nonsquamous PD-L1-positive NSCLC in selected population. *ACN* atezolizumab plus carboplatin and nab-paclitaxel; *CN* carboplatin and nab-paclitaxel; *PD-L1* programmed cell death-ligand 1; *NSCLC* non-small cell lung cancer

We identified 171 consecutive patients diagnosed with untreated metastatic nonsquamous PD-L1-positive NSCLC who had undergone the ACN or CN regimen, of whom 47 were excluded in accordance with the eligibility criteria. Thus, 124 eligible patients were evaluated. Among the 124 patients, 60 received the ACN regimen, and 64 received the CN regimen (Fig. 1). The patient characteristics are presented in Table 1. The median age was 64 years (range 46–75) in the ACN group and 63 years (47–72) in the CN group. In the ACN group, 68.3% of patients (*n* = 41) were male, 10.0% (*n* = 6) were never smokers, 60.0% (*n* = 36) had asymptomatic brain metastasis, and 78.3% (*n* = 47) had more than or equal to three metastatic sites. In the CN group, 52.8% of patients (*n* = 43) were male, 12.5% (*n* = 8) were never smokers, 57.8% (*n* = 37) had asymptomatic brain metastasis, and 75.0% (*n* = 48) had greater than or equal to three metastatic sites. ECOG-PS was 0 in 30.0% and 1 in 70.0% of individuals who underwent the ACN regimen versus 0 in 31.3% and 1 in 68.7% of patients who underwent the CN regimen (*p* = 0.881). PD-L1 tumour expression was TC1 in 25.0%, TC2 in 18.3%, and TC3 in 56.7% of patients who underwent the atezolizumab plus carboplatin and nab-paclitaxel regimen versus TC1 in 18.8%, TC2 in 21.9%, and TC3 in 59.3% of patients who underwent the carboplatin and nab-paclitaxel regimen (*p* = 0.708). The patient characteristics were well proportioned between the groups. At the final follow-up, 60 individuals in the ACN group and 64 in the CN group underwent at least one cycle of combination therapy. The median duration of combination therapy was similar between both cohorts (17 months [range 1–37] in the atezolizumab plus carboplatin and nab-paclitaxel regimen group and 16 months [1–36] in the carboplatin and nab-paclitaxel regimen group). No significant differences were detected in the number of administrations of nab-paclitaxel between both groups. The median cycles were 22 (range 1–28) in the ACN group and 21 (1–25) in the CN group.

**Table 1:** Baseline data of patients included in the study

Variable	ACN (*n* = 60)	CN (*n* = 64)	*p* value
Age (years), *n* (%)			0.773^a^
< 60	21 (35.0)	24 (37.5)	
≥ 60	39 (65.0)	40 (62.5)	
Sex, *n* (%)			0.892^a^
Male	41 (68.3)	43 (52.8)	
Female	19 (31.7)	21 (67.2)	
ECOG-PS, *n* (%)			0.881^a^
0*	18 (30.0)	20 (31.3)	
1**	42 (70.0)	44 (68.7)	
Time since diagnosis, month(s)	3 (1–6)	3 (1–7)	0.901^b^
PD-L1 tumour expression (TC)#, *n* (%)			0.708^a^
TC1 (1–5)	15 (25.0)	12 (18.8)	
TC2 (5–50)	11 (18.3)	14 (21.9)	
TC3 (≥ 50)	34 (56.7)	36 (59.3)	
Tobacco use history, *n* (%)			0.186^b^
Never	6 (10.0)	8 (12.5)	
Current	12 (20.0)	19 (29.7)	
Previous	42 (70.0)	37 (57.8)	
Brain metastasis, *n* (%)			0.871^a^
Asymptomatic	36 (60.0)	37 (57.8)	
Without	24 (40.0)	27 (42.2)	
Duration of treatment month (s)	17 (1–37)	16 (1–36)	0.437^a^
Number of metastatic sites, *n* (%)			0.663^b^
< 3	13 (21.7)	16 (25.0)	
≥ 3	47 (78.3)	48 (75.0)	

*ACN* atezolizumab plus carboplatin and nab-paclitaxel; *CN* carboplatin and nab-paclitaxel; *ECOG-PS* Eastern Collaborative Oncology Group performance status; *TC* tumour cells; *PD-L1* programmed cell death-ligand 1

*Fully active, able to carry on all pre-disease performance without restriction

**Restricted in physically strenuous activity but ambulatory and able to carry out work of a light or sedentary nature; #TC: 1% ≤ TC1 < 5%, 5% ≤ TC2 < 50%, TC3 ≥ 50%

^a^Mann–Whitney *U* test

^b^Independent samples *t*-test

### Efficacy

**Fig. 2:**
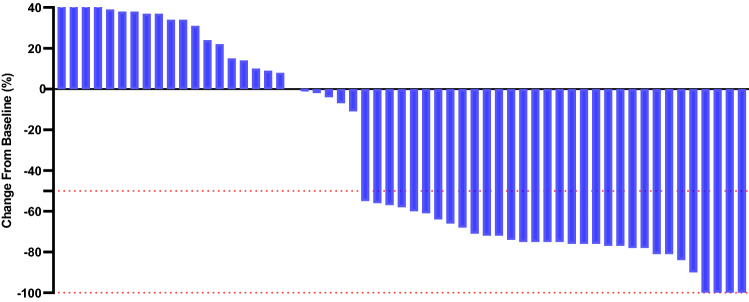
Percentage change from baseline in sums of diameters of target lesions by mRECIST in patients with metastatic nonsquamous PD-L1-positive NSCLC who experienced ACN regimen (*n* = 58)

**Fig. 3:**
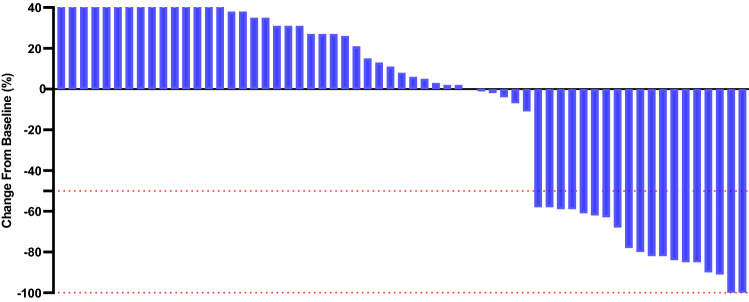
Percentage change from baseline in sums of diameters of target lesions by mRECIST in patients with metastatic nonsquamous PD-L1-positive NSCLC who experienced CN regimen (*n* = 61)

**Fig. 4:**
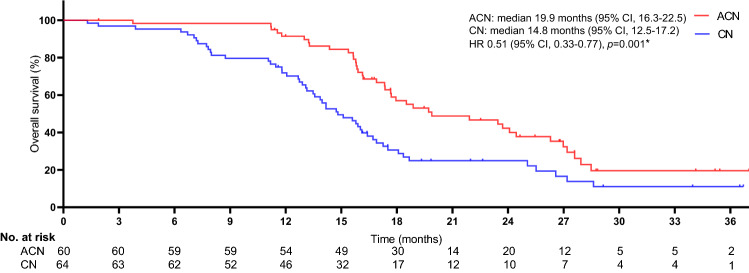
Kaplan–Meier curves for OS. The median OS was 19.9 months (95% CI 16.3–22.5) for ACN and 14.8 months (95% CI 12.5–17.2) for CN (HR 0.51, 95% CI 0.33–0.77; *p* = 0.001). *HR was estimated with a Cox proportional hazards model, with the age, sex, ECOG-PS, time since diagnosis, PD-L1 tumour expression, tobacco use history, brain metastasis, number of metastatic sites utilised as covariates, and intervention provided as the time-dependent variable. *OS* overall survival; *ACN* atezolizumab plus carboplatin and nab-paclitaxel; *CN* carboplatin and nab-paclitaxel; *HR* hazard ratio; *CI* confidence interval; *ECOG-PS* Eastern Collaborative Oncology Group performance status; *PD-L1* programmed cell death-ligand 1

**Fig. 5:**
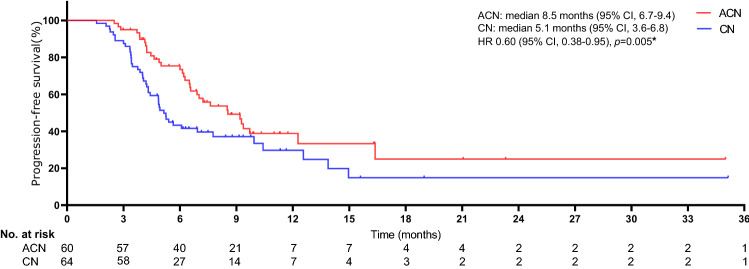
Kaplan–Meier curves for PFS. The median PFS was 8.5 months (95% CI 6.7–9.4) for ACN and 5.1 months (95% CI 3.6–6.8) for CN (HR 0.60, 95% CI 0.38–0.95; *p* = 0.005). *HR was estimated with a Cox proportional hazards model, with the age, sex, ECOG-PS, time since diagnosis, PD-L1 tumour expression, tobacco use history, brain metastasis, number of metastatic sites utilised as covariates, and intervention provided as the time-dependent variable. *PFS* progression-free survival; *ACN* atezolizumab plus carboplatin and nab-paclitaxel; *CN* carboplatin and nab-paclitaxel; *HR* hazard ratio; *CI* confidence interval; *ECOG-PS* Eastern Collaborative Oncology Group performance status; *PD-L1* programmed cell death-ligand 1

The median follow-up time was similar between treatment groups (27 months [range 1–37] in the atezolizumab plus carboplatin and nab-paclitaxel regimen group and 27 months [1–36] in the carboplatin and nab-paclitaxel regimen group). The objective response rate of 53.3% (95% CI 45.1–57.8) in the ACN group was higher than the 29.7% (26.1–32.8) observed in the CN group (*p* = 0.008) (Table 2). For patients in the ACN group, the ORR was 11.7%, 18.3%, and 23.3% for cohorts TC1, TC2, and TC3, respectively. For patients in the CN group, the ORR was 3.1%, 10.9%, and 15.6% for cohorts TC1, TC2, and TC3, respectively. The ORR tend to be lower in patients with low PD-L1 expression compared with those with high PD-L1 expression. Significant differences were detected in reductions in tumour size per independent imaging review by mRECIST (65.5% [38 of 58] in the ACN group vs. 40.9% [25 of 61] in the CN group, *p* = 0.008), as presented in Figs. 2 and 3. The 3-, 12-, and 24-month OS rates were 98.3%, 91.4%, and 42.2%, respectively, in the ACN group and 96.8%, 71.8%, and 24.9%, respectively, in the CN group. A noticeable distinction was seen in the median OS between treatment cohorts (19.9 months [95% CI 16.3–22.5] in the ACN group vs. 14.8 months [12.5–17.2] in the CN group), as presented in Fig. 4. The ACN regimen drastically improved the median OS compared to the CN regimen and resulted in a 49% lower risk of death than the CN regimen (HR 0.51; 95% CI 0.33–0.77; *p* = 0.001). A noteworthy distinction of 5.1 months in the median OS was seen, and the superiority of the ACN regimen over the CN regimen may be positive because a separation of survival curves was seen throughout the follow-up period. In addition, a noteworthy distinction in the median PFS was noted (8.5 months [95% CI 6.7–9.4] in the ACN group vs. 5.1 months [3.6–6.8] in the CN regimen group [HR 0.60; 95% CI 0.38–0.95; *p* = 0.005]), as exhibited in Fig. 5.

**Table 2:** Tumour response related to two regimens

	ACN (*n* = 60)	CN (*n* = 64)	*p* value
Best overall response, *n* (%)			0.006
CR	4 (6.7)	2 (3.1)	
PR	28 (46.7)	17 (26.6)	
SD	6 (10.0)	6 (9.4)	
PD	20 (33.3)	36 (56.3)	
Unclear	2 (3.3)	3 (4.7)	
Objective response rate*, *n* (%)	32 (53.3)	19 (29.7)	0.008

Tumour response was assessed by mRECIST per independent imaging review

*ACN* atezolizumab plus carboplatin and nab-paclitaxel; *CN* carboplatin and nab-paclitaxel; *CR* complete response; *PR* partial response; *SD* stable disease; *PD* progressive disease

*The proportion of confirmed CR or PR per independent imaging review

Subgroup analyses demonstrated that for individuals with TC3 PD-L1-expressing cells, the median OS was 23.7 months in the ACN group versus 16.1 months in the CN group (HR 0.25; *p* = 0.001); for individuals with TC2 PD-L1-expressing cells, the median OS was 19.1 months in the ACN group versus 15.3 months in the CN group (HR 0.39; *p* = 0.003); for individuals with TC1 PD-L1-expressing cells, the median OS was 16.8 months in the ACN group versus 12.6 months in the CN group (HR 0.12 months, *p* = 0.002).

### Safety

In total, 106 patients (85.5%) had one or more AEs related to first-line therapy. Of the 124 patients, interruption related to AEs occurred in 13 (10.4%) patients (6 of 60 individuals in the ACN group vs. 7 of 64 individuals in the CN group, *p* = 0.865). Key treatment-related grade ≥ 3 AEs are shown in Table 3. The safety profile of ACN was in accordance with the earlier described safety profile, without unfamiliar AEs detected. The key grade 3 or worse AEs related to first-line therapy were neutropenia (19 [31.7%] of 60 in the ACN group vs. 6 [9.4%] of 64 in the CN group) and anaemia (15 [25.0%] in the ACN group vs. 7 [10.9%] in the CN group). There were no noteworthy distinctions in regard to other grade ≥ 3 AEs. Deaths related to first-line therapy were not observed in the study.

**Table 3:** Treatment-related key ≥ grade 3 AEs in patients who underwent ACN or CN

Variable, *n* %	ACN (*n* = 60)	CN (*n* = 64)		HR (95%)	*p* value^a^
Neutropenia	19 (31.7)	6 (9.4)	0.46 (0.11–0.79)		0.002
Anaemia	15 (25.0)	7 (10.9)	2.16 (1.52–3.14)		0.041
Neutrophil count decreased	6 (10.0)	3 (4.7)	1.05 (0.69–2.84)		0.256
Thrombocytopenia	5 (8.3)	4 (6.3)	2.63 (0.74–3.15)		0.656
Platelet count decreased	4 (6.7)	2 (3.1)	1.72 (0.93–2.66)		0.360
Fatigue	4 (6.7)	2 (3.1)	1.31 (0.90–2.78)		0.360
Decreased white blood cell	3 (5.0)	3 (4.7)	2.01 (0.77–3.17)		0.936
Diarrhoea	2 (3.3)	2 (3.1)	1.21 (0.55–2.39)		0.948

*ACN* atezolizumab plus carboplatin and nab-paclitaxel; *CN* carboplatin and nab-paclitaxel; *HR* hazard ratio

^a^Mann–Whitney *U* test

## Discussion

The findings of this study demonstrate that the atezolizumab plus carboplatin and nab-paclitaxel regimen as front-line management might provide greater survival benefits in PD-L1-positive metastatic nonsquamous NSCLC patients in a selected population than the carboplatin and nab-paclitaxel regimen, with a tolerable safety profile. The high OS indicates that the treatment regimen incorporating atezolizumab with carboplatin and nab-paclitaxel might have synergistic effects and prevent the progression of tumours.

The study findings are consistent with the findings of a randomised, phase 3 trial (IMpower130) (West et al. 2019) that compared the clinical outcomes of atezolizumab plus carboplatin and nab-paclitaxel vs. carboplatin and nab-paclitaxel as front-line therapies in 723 individuals with pretreated advanced nonsquamous NSCLC. The results indicated that noteworthy improvements were detected in regard to median OS (18.6 months [95% CI 16.0–22.2] in the ACN group and 13.9 months [12.0–18.7] in the CN group) (HR 0.79; 95% CI 0.64–0.98; *p* = 0.033) and median PFS (7.0 months [95% CI 6.2–7.3] in the atezolizumab plus carboplatin and nab-paclitaxel regimen group and 5.5 months [4.4–5.9] in the carboplatin and nab-paclitaxel regimen group (HR 0.64; 95% CI 0.54–0.77; *p* < 0.0001)). This IMpower130 trial showed that the addition of atezolizumab to carboplatin and nab-paclitaxel is a promising anticancer option. Moreover, a trend towards excellent survival related to atezolizumab was noticed, irrespective of EGFR or ALK mutations. Similarly, a subgroup analysis of the phase 3 OAK trial (NCT02008227) (Hida et al. 2018) of 64 Japanese patients diagnosed with locally advanced PD-L1-positive NSCLC who underwent therapy with atezolizumab showed that a longer median OS was detected for the atezolizumab regimen than for the docetaxel regimen (21.3 months vs. 17.0 months, respectively; HR 0.80; 95% CI 0.41–1.57). In 2017, a phase 2 trial (Peters et al. 2017) of atezolizumab as a front-line or subsequent therapy for individuals with PD-L1-selected metastatic NSCLC (BIRCH) demonstrated that the median OS in an updated survival analysis of TC3 patients administered single-agent atezolizumab was 26.9 months; the median OS for patients administered atezolizumab combined with one prior platinum chemotherapy was 15.5 months; and the median OS for patients administered atezolizumab plus at least two prior chemotherapies was 13.2 months. In the BIRCH trial, prior cytotoxic chemotherapy did not appear to promote survival, and noteworthy OS improvement was detected in individuals with high TC3 PD-L1 expression.

Expression of PD-L1 in the tumour microenvironment tends to result in a poor prognosis (Borghaei et al. 2021; Gettinger et al. 2018; Gettinger et al. 2015; Haratani et al. 2018; Kazandjian et al. 2016). A high mutation rate related to NSCLC suggests that NSCLC cells may have a promising durability of response to immune checkpoint inhibitors (Borghaei et al. 2015; Gettinger et al. 2016; Owonikoko et al. 2021), although an association is lacking between tumour response and PD-L1 expression in the phase 1 trial of atezolizumab (Hellmann et al. 2020). Restoring tumour-specific T cell immunity using directly inhibiting PD-L1-PD-1 signalling has shown sustained anticancer activity in the management of PD-L1-positive NSCLC (Borghaei et al. 2021; Horn et al. 2018; Ikeda et al. 2021). Combining atezolizumab with carboplatin and nab-paclitaxel may have synergistic antitumour effects related to immune microenvironment modulation and might reduce or avoid the occurrence of immune escape and improve anticancer effects (Reck et al. 2020; West et al. 2019). An open-label phase IB study(Liu et al. 2018) of 26 patients with untreated stage IIIB/IV NSCLC who were administered atezolizumab plus carboplatin and nab-paclitaxel for four to six cycles, followed by atezolizumab maintenance, showed that the incorporation of atezolizumab into the carboplatin and nab-paclitaxel regimen was promising with a response rate of 46%, median OS of 17.0 months (95% CI 12.7–not evaluable), and median PFS of 5.7 months (95% CI 4.4–14.8).

Although individuals with PD-L1-positive NSCLC have a greater OS benefit attributed to atezolizumab, PD-L1 expression alone seems to partially explicate the OS benefit in individuals with PD-L1-positive NSCLC receiving atezolizumab plus cytotoxic chemotherapy for front-line management (Chalabi et al. 2020; Mansfield et al. 2020). Although NSCLC initially reacts strongly to cytotoxic chemotherapy, characterised by a substantial reduction in tumour burden, NSCLC often recurs or becomes resistant to chemotherapeutic drugs during or after treatment (Hopkins et al. 2021; von Pawel et al. 2019). The combination of VEGF-mediated immunomodulatory effects and chemotherapy-induced tumour cell antigen exposure may have a good synergistic anticancer effect (Ettinger et al. 2019; Reck et al. 2020). Stimulation of VEGF-mediated immunoregulation might be initiated by EGFR/ALK signalling (Peters et al. 2017). The enhancement of the anticancer immune response may be strongly associated with cytotoxic chemotherapy-induced exposure of tumour antigens (Reck et al. 2020). Although the IMpower130 trial (West et al. 2019) demonstrated that atezolizumab combined with carboplatin and nab-paclitaxel had a better OS benefit than carboplatin and nab-paclitaxel in front-line management of treatment-naïve, nonsquamous NSCLC in the wild-type population, the trial eliminated individuals with EGFR/ALK mutations and/or liver metastases and offer negligible survival benefit to these cases. A previous IMpower150 trial (Hopkins et al. 2021) compensated for these shortcomings, and the results from the trial further confirmed that the combination has anticancer activity in nonsquamous NSCLC.

The present study has several drawbacks. First, the retrospective design is associated with inherent biases. In the case of uncontrolled confounding, we cannot be sure of the magnitude of the effects we observed. Data with a high probability of misuse may be unavoidable and may have affected our results. Furthermore, regarding confusion issues, although we adopted strict inclusion criteria and checked all patients for potential diseases, these were at best only partial measures, and confusion issues may still have affected the analysis results. Second, during the follow-up period, the aetiology analysis of some deaths was not detailed. When difficulties in interpretation occurred, our diagnosis was cancer-related death, which may lead to uncertainty in the outcome because some patients may have died due to suicide or accident. In addition, when the follow-up data for individual patients were unclear or unavailable, indirect acquisition was used, which may have had an impact on the results. Third, the collection of patient baseline data was limited to the content recorded in the electronic health record. Errors may lie in patient memories and clinician records. Fourth, the generalizability of this study was limited to selected populations of Chinese patients diagnosed with treatment-naïve stage IV nonsquamous PD-L1-positive NSCLC.

## Conclusion

The results from this review support the extending body of evidence indicating that combining atezolizumab with carboplatin and nab-paclitaxel chemotherapy might have encouraging survival benefits in patients with treatment-naïve metastatic nonsquamous PD-L1-positive NSCLC, with a tolerable safety profile. Given the retrospective nature of this study, we cannot draw any definite conclusions.

## Data Availability

All data generated or analysed during this study are included in the article.

## Abbreviations

PD-L1: Programmed death-ligand 1
PD-1: Programmed cell death 1
NSCLC: Non-small cell lung cancer
OS: Overall survival
PFS: Progression-free survival
AEs: Adverse events
CI: Confidence interval
HR: Hazard ratio
VEGF: Vascular endothelial growth factor
EGFR: Epidermal growth factor receptor
ALK: Anaplastic lymphoma kinase
TCs: Tumour cells
ICs: Immune cells
mRECIST: Modified response evaluation criteria in solid tumours
